# Major Neutrophilia Observed in Acute Phase of Human Leptospirosis Is Not Associated with Increased Expression of Granulocyte Cell Activation Markers

**DOI:** 10.1371/journal.pone.0165716

**Published:** 2016-11-01

**Authors:** Loic Raffray, Claude Giry, David Vandroux, Barbara Kuli, Andry Randrianjohany, Anne-Marie Pequin, Frédéric Renou, Marie-Christine Jaffar-Bandjee, Philippe Gasque

**Affiliations:** 1 Université de La Réunion, CNRS 9192, INSERM U1187, IRD 249, CHU de La Réunion, Unité Mixte Processus Infectieux en Milieu Insulaire Tropical (PIMIT), Plateforme Technologique CYROI, Sainte-Clotilde, La Réunion, France; 2 Laboratoire d’immunologie clinique et expérimentale ZOI (LICE OI), CHU La Réunion site Félix Guyon, St Denis, La Réunion, France; 3 Internal Medicine Unit, CHU La Réunion site Félix Guyon, St Denis, La Réunion, France; 4 Microbiology/Virology Laboratory, CHU La Réunion site Félix Guyon, St Denis, La Réunion, France; 5 Intensive Care Unit, CHU La Réunion site Félix Guyon, St Denis, La Réunion, France; 6 Infectious Diseases Unit, CHU La Réunion site Félix Guyon, St Denis, La Réunion, France; 7 Internal Medicine Unit, GHER Hospital, St Benoit, La Réunion, France; 8 Hematology laboratory, CHU La Réunion site Félix Guyon, St Denis, La Réunion, France; Cornell University, UNITED STATES

## Abstract

It has long been known that pathogenic *Leptospira* can mobilize the immune system but the specific contribution of neutrophils to control the infectious challenge remains to be clarified. We herein analyzed the phenotype of circulating neutrophils of patients with leptospirosis and healthy controls for the expression of toll-like receptor (TLR) type 2 (TLR2, to sense the leptospiral LPS) and several activation markers: interleukin 8 chemokine receptor CD182 (CXCR2), CD11b of the integrin/opsonin complement receptor type 3 (CR3) and CD15 (ligand of the selectin). The plasmatic level of the main CD182 ligand, interleukin 8 (CXCL8), was measured by ELISA. Hospitalized leptospirosis cases showed marked neutrophilia, particularly in the most severe cases. Interestingly, TLR2 was significantly increased in leptospirosis but identical levels of CD182 and CD11b were detected when compared to controls. CD15 was significantly decreased on neutrophils in leptospirosis but returned to normal within 1 month. Basal levels of IL-8 were measured in control subjects and were not increased in leptospirosis cases at the initial stage of the disease. In conclusion, we observed that neutrophils failed to regulate the expression of several of the receptors involved in cell activation and recruitment. This study further emphasizes the paradigm that neutrophils may be impaired in their overall capacity to thwart bacterial infection in leptospirosis patients.

## Introduction

Leptospirosis is a worldwide infectious disease caused by *Leptospira* species, with a recent estimate of 1 million cases per year [1]. This spirochaetal zoonosis is potentially life-threatening with mortality rates ranging from 5% to 15% [1,2]. The course of the disease includes a broad spectrum of manifestations, encompassing asymptomatic or influenza-like illness to multi-organ failure with icteric hepatitis, acute renal failure and intra-alveolar hemorrhage notably [2]. Although detrimental complement evasion is increasingly documented [3], there are relatively few data regarding the role of innate immune phagocytic cells such as polymorphonuclear neutrophils (PMN).

During the first two weeks of leptospirosis, PMN count is usually characterized by a moderate increase and is correlated to disease severity [4–6]. This is also a non-specific feature of acute sepsis and bacteremia in general. PMN may act against *Leptospira* using soluble factors like antimicrobial peptides or oxidative stress [7–9]. The production of reactive oxygen species (ROS) seems to be more elevated in leptospirosis patients compared to controls, and is correlated to levels of markers of tissue injury, although the source of ROS and the contribution of PMN was not evaluated [10]. Recent findings suggest that the production of neutrophil’s extracellular traps (NETosis) could be a reliable mechanism of defense to prevent bacterial dissemination [11]. Beside these weapons, pathogenic leptospires are able to evade the immune response of PMN. A study has shown that the pathogenic strains are adherent to the granulocyte cell surfaces but are barely phagocytized by PMN [12]. In addition, *Leptospira* is able to exploit pyruvate to rescue H_2_O_2_ killing *in vitro*, and avoiding H_2_O_2_ killing [13]. A clearer understanding of the role of PMN during leptospirosis warrants further studies.

After the initial phase of bacteremia, *Leptospira* invades several organs, including liver, kidneys and lungs. Recruitment of PMN in inflamed tissues is a complex and coordinated sequence of events mediated by multiple soluble and cellular factors such as chemokines, selectins and integrins [14,15]. A tight regulation of PMN migration is mandatory to permit pathogen elimination in the inflamed tissues. While few studies have indicated that endothelial cells can be activated in leptospirosis [16–18], little is known about the capacity of PMN to be activated and mobilized from the circulation to adhere to vessels and to infiltrate target organs.

Canonically, during sepsis and septic shock mediated by Gram-negative bacteria, one major chemokine implicated in PMN chemotaxis is interleukin 8 (CXCL8), and its receptor, CD182 (CXCR2), is critical for the recruitment of PMNs [19,20]. During infection, CXCL8 is up-regulated at the site of inflammation, allowing the immune system to direct PMN in inflamed tissues via CD182 [21]. However, in severe sepsis, in humans and animal models of septic shock, it has been shown that the expression of CD182 was decreased, possibly altering the recruitment of PMN [22,23]. This down-regulation of CD182 may be explained by its internalization in the presence of high levels of circulating CXCL8, but it can also be a consequence of TLR2 stimulation as demonstrated in several reports [24,25]. Strikingly, TLR2 is the obligate receptor of LPS from *Leptospira* [26]. To date, the levels of the cellular molecules (markers) implicated in PMN response to LPS (TLR2) and cell activation (CD11b, CD15 and CD182) to mediate a robust innate immune response have not been studied in the setting of acute leptospirosis in humans. This was the main objective of this study.

## Material and Methods

### Cohort study and ethics

Our study was conducted in a medical center of the University Hospital of Reunion Island: 13 healthy subjects and 15 patients with confirmed leptospirosis (by PCR or serology) were enrolled. Patients were hospitalized in the intensive care unit or conventional medical units. They were treated according to the standards of care. Clinical and laboratory data were recorded until the point of discharge or death.

Definitions of the disease hallmarks:

- Severe leptospirosis was defined as a disease associated with severe organ injury and corresponded to patients fulfilling at least one of the following criteria: either jaundice (bilirubin > 50 μmol/L), aspartate aminotransferase increase (> 3 fold the upper normal limit), acute renal failure with creatinine clearance inferior to 30 ml/min (MDRD) or requirement of hemodialysis, mechanical ventilation or oxygen requirement, hypotension requiring fluid resuscitation.
- Leptospirosis stages: patients were enrolled during the first days after onset of symptoms and were defined as acute phase or M0 (month 0). Biological and immunological evaluations were also performed 1 month later after discharge of the patient and defined as convalescent phase or M1 (month 1).

Healthy controls (workers at the hospital) were matched with patients for age and sex. This study was conducted according to the principles expressed in the Declaration of Helsinki and was approved by the local human ethic committee of “CHU de La Réunion” (number R15018). All patients provided written informed consent for the collection of samples and subsequent analyses were performed anonymously.

### Real-time quantitative PCR analyses for diagnosis and quantification of leptospirosis

Biological specimens for diagnosis of leptospirosis were sampled at admittance of patients. Leptospires in plasma or urine were detected by quantitative real-time PCR (qPCR) using the Light cycler LC480 system (Roche), and TaqMan® Universal PCR Mastermix with primers and probe specific for 23S rRNA gene of *Leptospira* as detailed before [27]. For quantification of bacterial burden in plasma by PCR, serial dilutions of genomic DNA extracts from *L*. *interrogans* serogroup Icterohaemorragiae serovar Copenhageni were performed. These dilutions corresponded to concentrations from 4 x 10^6^ to 4 bacteria/ml and the number of bacteria per ml in plasma samples were inferred from the cycle threshold (Ct) values of PCR according to the log-transformed standard curve, as detailed in previous reports [28].

### Serological testing for leptospirosis

For patients with a strong suspicion for acute leptospirosis but negative by PCR in blood or urine samples, we have performed serological testing. If the detection of anti-leptospiral IgM antibodies by ELISA (SERION) was positive (>50 U/mL), the testing was completed with a micro-agglutination test (MAT) performed by the national reference centre of Pasteur Institute in Paris. Patients were considered positive above a MAT titer of 1/400. The MAT was also used to identify the serovars in several patients after the convalescent phase.

### Flow cytometry analyses

Peripheral blood was collected in EDTA vacutainer tubes from 13 healthy controls and 15 patients with acute leptospirosis confirmed by PCR or serology. Within 6 hours after sampling on tubes containing ethylenediaminetetraacetic acid (EDTA), 100 μL of whole blood was mixed with 5 μL monoclonal antibodies against different cell surface markers. The following antibodies were used: isotypic controls coupled with FITC (fluorescein isothyocyanate, Biolegend 400108) and PE (phycoerythrin, Biolegend 400212), anti-CD16-ECD (Beckman Coulter A33098), anti-CD182-FITC (Biolegend 320704), anti-CD11b-FITC (Beckman Coulter IM 0503), anti-CD15-PE (Biolegend 323006) and anti-TLR2-PE (Biolegend 309708). Tubes were vortexed after adding each antibody. After 30 minutes of incubation at room temperature in the dark, red blood cells were lysed with Beckman ImmunoPrep™ Reagent System (Beckman Coulter ref 7546999) using TQ-Prep™ Workstation. Cells surface markers data were acquired by flow cytometry (Beckman Coulter, Navios™ Cytometer and Navios™ acquisition software, version 1.2) and analyzed with Kaluza® Analysis Software version 1.3 (Beckman Coulter™). PMN were gated according to FSC and SSC characteristics, and then CD16+ cells were considered for further analysis. The mean fluorescence intensity (MFI) values are evaluated after adjusting cytometer to obtain MFI of isotypic controls to 10^−1^.

### ELISA

Interleukin 8 (CXCL8) chemokine levels were measured in plasma from healthy subjects and leptospirosis patients. Plasma were conserved at -80°C and tested by ELISA using the kit from eBioscience™ (Human ELISA Ready-Set-Go ref. 88-8086-88).

### Statistics

Data are expressed as medians and interquartile ranges for quantitative variables; and as numbers and percentages for qualitative variables. Owing to non-Gaussian distribution, statistical significance of difference between groups was determined by non-parametric Mann-Whitney U-test for continuous variables and by Khi-2 square test for qualitative variables. For paired data the Wilcoxon non-parametrical test was used. The Spearman test was used to analyze correlations among variables. *P*-values below 0.05 were considered statistically significant. Statistics were performed with GraphPad Prism™.

## Results

### Population study

Patients were included in the study prospectively between February and June 2015, during the rainy season in Réunion Island. Among the 15 hospitalized patients, 14 were male and the median age was 46.8 years (IQR = interquartile range: 28.2–61.4), see **Table 1**. The source of contamination was recreational activities for 9 patients: bathing in rivers (n = 5), gardening (n = 3) or hunting (n = 1). The 6 patients exposed to professional risks were farmers (n = 4) or green space workers (n = 2). Seven patients had at least one additional risk with residential exposure to rats in the neighborhood, or living in a rural area. All patients had influenza-like illnesses or isolated fever as a first sign of illness. The median time frame to hospitalization was 5 days (ranges 1–7). Patients presented with markers of classical organ damages: jaundice (n = 6), acute renal failure (n = 8), and rhabdomyolysis with creatinine kinase >1000 UI/L (n = 9). Patients presented also thrombocytopenia (<100.10^9^/L) (n = 10). The values of biological data recorded at admittance were significantly different from the healthy subject controls (Table 1), and systematically associated to the more severe cases of leptospirosis (n = 10) compared to milder forms (n = 5). Among the 10 severe forms, 9 patients were hospitalized in intensive care unit as they required dialysis (n = 6), mechanical ventilation (n = 3) and/or vasopressor drugs support (n = 3). Despite receiving antibiotics, 1 patient died because of septic shock complicated by multiorgan failure within 48 hours after admittance. All other patients received antibiotic course, mainly amoxicillin, and had a favorable outcome after median stay in hospital of 6 days (ranges 2–25).

**Table 1:** Characteristics of leptospirosis patients group at admittance and comparison to controls.

Characteristic (units)	Controls	Leptospirosis cases	Mild leptospirosis	Severe leptospirosis	*P* value	
					controls vs. lepto	mild vs. severe
**Number of individuals**	13	15	5	10		
**Ratio M/F**	12/1	14/1	5/0	9/1	NS	NS
**Age (years)**	34.1 (25.8–48.6)	46.8 (28.2–61.4)	46 (24.6–46.8)	52.5 (28.6–61.9)	NS	NS
**Neutrophils (10**^**9**^**/L)**	3.4 (2.6–3.8)	8.9 (7.4–9.8)	9 (8.9–9.2)	8.7 (7.3–11.6)	<0.0001	NS
**Lymphocytes (10**^**9**^**/L)**	2.0(1.9–2.7)	0.62 (0.53–0.92)	0.69 (0.56–0.84)	0.59 (0.42–0.91)	<0.0001	NS
**Monocytes (10**^**9**^**/L)**	0.48(0.35–0.64)	0.61 (0.39–0.74)	0.65 (0.61–0.76)	0.52 (0.38–0.71)	NS	NS
**Platelets (10**^**9**^**/L)**	236 (226–260)	50 (34–103)	101 (95–194)	37.5 (30–49)	0.0001	0.02
**Creatinine (**μ**mol/L)**	84 (81–91)	151 (99–341)	103 (96–112)	282 (115–399)	0.001	NS
**Total bilirubin (**μ**mol/L)**	11 (9–12)	41 (25–89)	16 (14–23)	53 (41–126)	0.0002	0.005
**AST (IU/L)**	26 (18–31)	70 (39–146)	39 (36–56)	97 (66–198)	0.0003	0.049
**CPK (IU/L)**	128 (106–216)	1273 (845–2378)	714 (324–1080)	1960 (1264–4911)	0.0013	NS
**CRP (mg/L)**	0.5 (0.4–2)	194 (187–215) (n = 9)	194 (151–215)	203 (192–256) (n = 4)	0.0001	NS
Plasma bacterial load* **(bact./mL)**	NA	262 (67–1560) (n = 12)	52 (24–163) (n = 4)	1002 (92–2689) (n = 8)	NA	0.04

Data are expressed as medians (interquartile ranges). Statistics between 2 groups are performed with nonparametric unpaired tests (Mann-Whitney U-test) for quantitative variables, and with Khi-2 square test for categorical data. P value inferior to 0.05 was considered significant.

*Plasma bacterial load is inferred from plasma PCR values according to the log-transformed standard curve.

(AST = aspartate aminotransferase; CPK = creatinine phosphokinase; CRP = C-reactive protein; NS = not significant)

**Fig 1:**
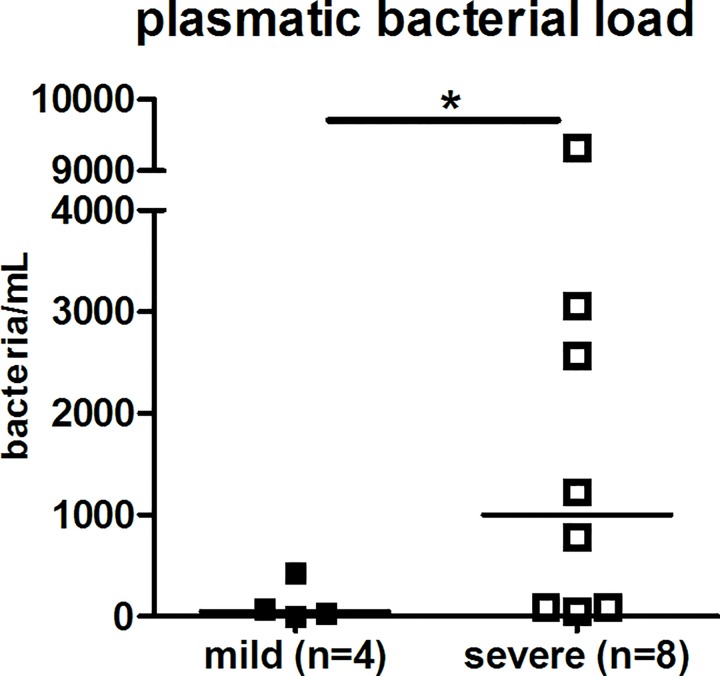
Bacterial burden is higher in severe leptospirosis. Plasma bacterial load is established from the plasma PCR values (n = 12) and using the log-transformed standard curve as described [27]. Horizontal bars indicate the median. Comparison with non-parametric Mann-Whitney test. * indicates P-value inferior to 0.05.

The diagnostic was established by plasma real-time PCR for 12 patients, urine PCR analysis for 2 patients, and serological testing for 1 patient (IgM+) for whom PCR were negative. One patient was positive by PCR in blood and urine samples. Of note, the bacterial burden established from PCR data was more elevated in the severe group: P = 0.04 (**Fig 1**). For 9 convalescent individuals, a complementary analysis was performed 1 month after hospital discharge (M1 patients) in order to identify the *Leptospira* species implicated. The MAT screening at M1 indicated infection consecutive to *L*. *Icterohaemorragiae* in 5 cases, while it was impossible to conclude in 4 cases, as a consequence of test positivity (titer>1:400) associated to multiple serovars: *L*. *Icterohaemorragiae*, *L*. *canicola*, and one for *L*. *ballum*.

### Unique neutrophilia during the course of leptospirosis in patients

**Fig 2:**
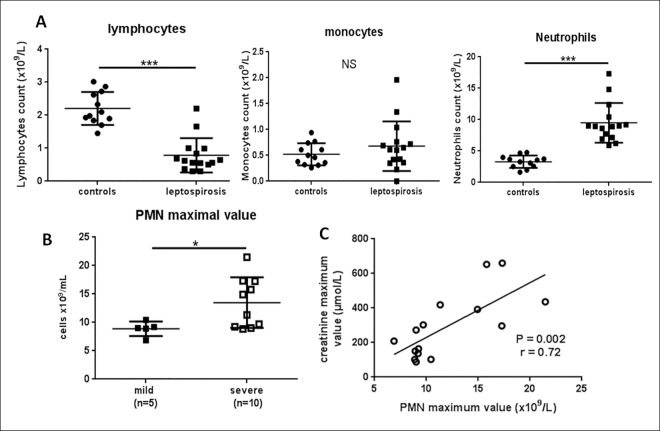
Neutrophil levels at admittance and during the disease course of leptospirosis patients. (A) Counts of immune cells for healthy controls (circles) and leptospirosis patients (squares) at admittance (M0). (B) The maximal value of PMN count reached during hospital stay was compared between mild and severe forms. For A and B, the largest horizontal bars indicate the median value, upper and lower bars the interquartile ranges. Comparisons with non-parametric Mann-Whitney test. *, *** indicate P-value inferior to 0.05 and 0.0001 respectively. (C) Among the patients with leptospirosis, the maximal value of PMN was correlated to the maximal value of creatinine with Spearman test.

The quantification of circulating immune cells at admittance (M0) showed marked lymphopenia, no significant change in monocytes count, while there was a significant increase in PMN cells to 8.9 x10^9^/L on average (7.4–9.8) in patients with leptospirosis, **Fig 2A**. Notably, there was no difference between severe and mild forms regarding PMN counts determined at admittance (**Table 1**). We further analyzed PMN count and possible links with clinical hallmarks. There were no correlations between PMN counts in leptospirosis patients with either age, duration of hospital stay, or number of damaged organs. Along the same lines, PMN count was not linked to the levels of the markers associated with organ injury such as AST, bilirubin, platelets, creatinine or creatinine phosphokinase levels. Moreover, PMN count was not statistically correlated to plasma bacterial load at admittance for 12 of leptospirosis patients.

In contrast, bacterial load was found to be positively correlated to the length of hospital stay (P = 0.03, r value at 0.8 with Spearman test) or the level of bilirubin (P = 0.03 and r = 0.64) and negatively correlated to the platelet counts (P = 0.02, r = 0.36).

Given that the duration of the disease prior to hospitalization was different from one patient to another, we also performed analyses with the maximal values of PMN counts during the disease course. The maximal values of PMN were at a median of 9.7 x10^9^/L (9–15.4), and these values were observed in median at the second day after admittance (IQR 1–4). This corresponded to the 7^th^ day (6–9) from the onset of symptoms (fever and myalgia).

The severe forms had a greater increase in PMN counts (P = 0.04) when considering the maximal value of PMN during hospitalization (**Fig 2B**). Moreover, the maximal value of PMN count presented a significant correlation with the maximal value of creatinine: r = 0.72, P = 0.002 (**Fig 2C**).

### Expression of TLR2 and markers of cell activation on PMN in leptospirosis

**Fig 3:**
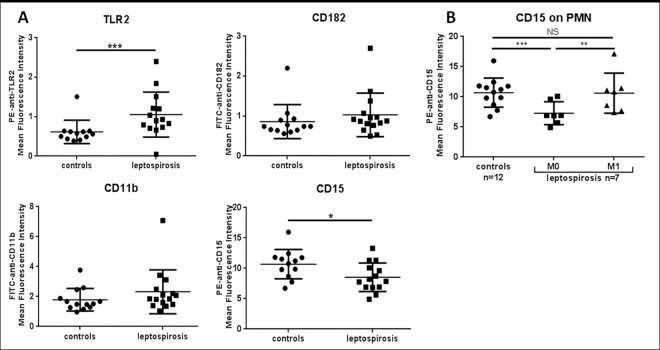
Expression of cell surface molecules implicated in PMN recruitment and activation during leptospirosis. (A) Expression levels of receptors and ligands on circulating PMN at admittance, assessed by flow cytometry. The mean fluorescence intensity (MFI) values were evaluated while isotypic negative controls were set with an MFI of 10^−1^. One value is missing for CD15. (B) Evolution of CD15 expression among 7 leptospirosis patients and comparison between admittance (M0) and at 1-month (M1) post infection. Largest horizontal bars indicate the median value, upper and lower bars the interquartile ranges. Comparison of the patient group and healthy donor group with non-parametric Mann-Whitney test, and comparison within patients between M0 and M1 with non-parametric Wilcoxon paired test. *, **, *** indicate P-value inferior to 0.05, 0.001 and 0.0001 respectively.

Next, we investigated the expression of selected PMN cell surface molecules implicated in LPS sensing, and cell activation (chemotaxis, adhesion and phagocytosis) for patients in the acute phase of the disease (M0) (**Fig 3A)**.

Focusing on TLR2 expression levels on PMN, we observed a significant increase in the leptospirosis group (P = 0.001). The MFI values were 0.92 (0.75–1.21) versus 0.57 (0.48–0.63) in healthy controls.

In contrast, we found no differences between controls and leptospirosis cases at M0 regarding CD182 and CD11b surface levels on PMN identified by the CD16 counterstaining. Concerning CD15, the expression in leptospirosis group was decreased compared to controls.

The changes in the expression levels of CD182, CD11b, CD15 and TLR2 were not correlated to any of the clinical and biological markers of disease and tissue injuries. The levels of expression of these receptors and PMN counts were further obtained from convalescent patients evaluated at 1 month after discharge (M1). The median value of PMN decreased at M1: 4.7 x10^9^ cells/L (4.1–6.1) compared to 8.9 x10^9^ cells/L (7.5–10.3) at M0 (P = 0.04). The levels for CD182 and CD11b between M0 and M1 remained identical. The level of CD15 expression on PMN at 1 month was significantly higher compared to the acute phase of leptospirosis: P = 0.02 with non-parametrical Wilcoxon test for paired data, **Fig 3B**. Of note, the MFI values at M1 post infection were not significantly different from the healthy controls and indicating a return to basal levels of expression for all PMN markers analyzed in our study.

**Fig 4:**
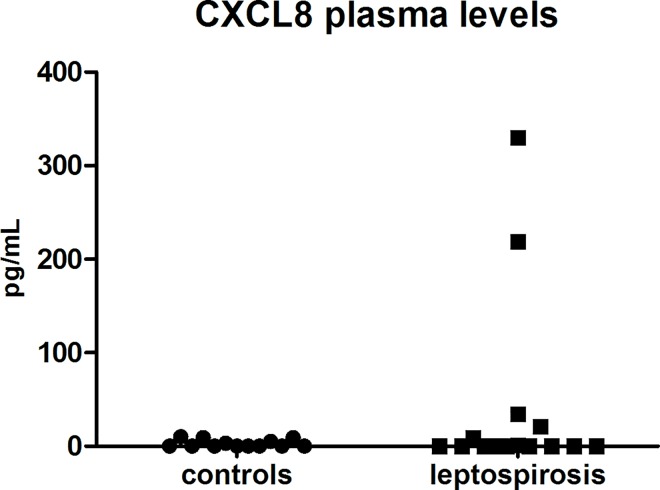
Circulating CXCL8 (interleukin 8) levels are not significantly elevated in leptospirosis cases. CXCL8 levels were assessed by ELISA of plasma from healthy control individuals (circles, n = 13) and leptospirosis cases (squares, n = 15). For leptospirosis cases, assessment was performed with sampling at hospital admittance corresponding to acute phase (M0), with PCR positive patients. Comparison between two groups with non-parametric Mann-Whitney U-test showed no significant difference.

Finally, we investigated the levels of CXCL8 in plasma of the 15 patients and 13 healthy controls. The chemokine was barely detectable (<10 pg/mL), except for four of the severe leptospirosis cases with values of 21, 34, 219 and 330 pg/mL (**Fig 4**). Three of these patients had septic shock with multiorgan failure and one of them died.

## Discussion

Innate immune cells such as PMN play a critical role to control bacterial infection. In this study we showed that the expression of several cell surface PMN molecules, implicated in chemotaxis and recruitment to inflamed tissues, were either not upregulated when compared to healthy subject controls, or decreased. Moreover, CXCL8, a major chemokine for leukocyte recruitment was barely elevated in leptospirosis. Counterintuitively, only four patients with severe forms of the disease had elevated levels of CXCL8.

To our knowledge, this is the first study assessing the capacity of circulating PMN to be activated during the acute phase of leptospirosis in humans. With the limits of partial exploration of the complex process implicated in the chemotaxis, adhesion and phagocytosis of PMN, our results indicate unique behavior between leptospirosis patients and those with classical Gram-negative multiple organ infection, as discussed below.

The studied population presented mild and severe forms of leptospirosis. Cases were confirmed by PCR or IgM serological assay. Although the number of individuals included in the study is limited, this number is sufficient to evaluate significant variations in cell counts and cell immunophenotyping.

The observed neutrophilia during the acute phase of leptospirosis was in agreement with previous reports. Moderate increase of PMN counts have been reported in patients with *Leptospira* and particularly in the most severe forms [4–6]. Neutrophilia has also been found in other Gram-negative bacterial infection and to control the infectious challenge and to limit tissue damage [29,30].

Interestingly, we found that this neutrophilia in blood sampled at admittance (M0 stage) was not correlated to the level of major organ injuries either at the clinical or biological levels. In contrast, a significant association was observed between the maximal PMN count and the maximum creatinine level during the disease course of leptospirosis.

Interestingly, the levels of CXCL8 was low in our leptospirosis cohort and contrasting to data published for classical Gram-negative severe sepsis cases [19]. CXCL8 was increased only in 4 patients and in the context of the more severe forms of the disease. Literature data have also indicated low levels of CXCL8 in human leptospirosis [31] but several other reports demonstrated increased levels and correlation to severity and mortality [32–34]. How can we possibly reconcile such discrepancies? The differences may be a consequence of different infecting serovars and this information is unfortunately lacking in many of the published studies. Another explanation may be that CXCL8 levels may be fluctuating during the disease course. Papa et al. recently demonstrated that CXCL8 peaked at 6–10 days post-infection [35]. In our study we sampled CXCL8 levels at 5 days post-infection in median. It would be interesting to study whether other chemokines are differentially expressed in leptospirosis. Experiments along these lines are now highly warranted.

TLR2 and TLR4 regulate important PMN functions such as release of chemokines, production of reactive oxygen species (ROS), and activation of major proinflammatory signaling pathways [36]. The expression levels of cell surface TLR2 and TLR4 on PMN is usually up-regulated upon stimulation by bacterial products, LPS or lipopeptides, as well as during sepsis [36,37]. TLR expression levels on PMN during infection by *Leptospira* have never been reported. It has been demonstrated that LPS of *Leptospira* could signal through murine TLR4 and TLR2 but that human TLR2 is the major receptor for leptospiral LPS [26,38]. Noticeably, it has been shown that a specific polymorphism in human TLR2 gene was associated with an increased risk of leptospirosis and influencing its severity [39]. Hence, we focused on TLR2 expression and showed that it was significantly up-regulated during leptospirosis. It has been found that TLR2 is upregulated on endothelial cells in lung samples of fatal leptospirosis [40]. TLR2 stimulation by LPS should induce CXCL8 synthesis and secretion at least *in vitro* [36] and yet this was not detected in our patients. This would indicate that the innate immune response of circulating PMN in the presence of LPS from *Leptospira* is tightly regulated.

With regards to the activation status of PMN, this work indicates no major changes in the expression of CD182, CD11b while CD15 expression was decreased. The number of receptors studied is limited and other key effectors molecules should also be considered and studied. The membrane-bound receptors studied are nevertheless representative of the main steps of the PMN adhesion cascade to an activated endothelium [15,21]. It remains to be ascertained whether the levels of LFA-1 (integrin CD11a/CD18) or PSGL1, the P-selectin glycoprotein ligand 1 are affected on PMN during the course of leptospirosis. CD15 is a glycoprotein that binds to the major P-, E- and L-selectins indispensable for the rolling step of PMN[41]. Data from other studies regarding CD15 expression in the setting of sepsis are scarce but clearly indicated a basal or increased CD15 expression [42,43]. Thus, the decrease of CD15 level in leptospirosis cases might reflect a poor capacity of circulating PMN to be recruited to the tissues infected by *Leptospira*. CD11b (alpha subunit of CR3) is crucial for the slow rolling, adherence and transendothelial migration towards inflamed tissues as well as phagocytosis of complement opsonized-bacteria [15]. During the coordinated response to canonical bacterial sepsis, PMN activation is accompanied by CD11b up-regulation [44–46]. Moreover, infection by the spirochete *Borrelia burgdorferi* demonstrated an activation state of PMN with an increase of CR3 expression [47]. In our study we did not observe such increase. This unique observation adds to the plausible impaired innate immune function of PMN in leptospirosis.

CD182 is a receptor for several important CXCL chemokines, including CXCL8, and it induces activation of PMN during the rolling step next to an activated endothelium. As mentioned previously, sepsis is usually associated with CD182 down-regulation [22,23,45], a mechanism thought to participate to immune paralysis during sepsis and septic shock. This down-regulation may be induced either by high amounts of CXCL8, the most potent ligand of CXCR2 or by TLR2 stimulation as demonstrated by Alves-Filho et al [24]. This feed-back loop would allow PMN to respond to high CXCL8 gradient released by the vasculature and in order to invade inflamed tissues [21]. Of note, CXCL8 and CD182 are not the only ligand/receptor to engage PMN chemotaxis and lower expression of CD182 is not inevitably associated to chemotaxis impairment as demonstrated by Sabroe et al [25]. As a note of caution, we have studied the phenotype of circulating PMN at a given time of the disease process (acute phase of the disease-M0- and in convalescent phase–M1) and longitudinal studies are now warranted. Interestingly, the levels of PMN cell markers returned to control basal levels at M1 post infection. The activation status of PMN can change greatly between bloodstream and inflamed tissues [48] and despite the difficulties in performing such studies in humans, experiments along these lines are warranted.

## Conclusion

Our study provides unique information regarding the phenotype and possible functional impairments of PMN during the process of *Leptospira* infection. In sharp contrast to the paradigm reported in Gram-negative sepsis, CD182 expression remained at basal levels, CD11b expression did not increase and CD15 level was down regulated. Taken together our novel observations question the ability of PMN to be mobilized into inflamed target-tissues during leptospirosis; PMN migration and phagocytic functions may also be impaired. Further studies should be performed to address the expression of other receptors essential for PMN’s mobilization as well as performing functional phagocytic assays in the presence of *Leptospira*.
